# Clinical value of esketamine combined with ropivacaine in rebound pain after brachial plexus block in patients with upper limb fractures

**DOI:** 10.3389/fsurg.2024.1470205

**Published:** 2024-11-27

**Authors:** Shiyao Zhu, Dan Wang, Haiyan Gao, Lei Heng, Weikang Shui, Shanshan Zhu

**Affiliations:** ^1^ Department of Anesthesiology, Xuzhou Cancer Hospital, Xuzhou, China; ^2^ College of Anesthesiology, Xuzhou Medical University, Xuzhou, China

**Keywords:** brachial plexus block, esketamine, rebound pain, ropivacaine, upper limb fractures

## Abstract

**Objective:**

To analyze the clinical value of the combination of esketamine and ropivacaine in alleviating rebound pain in patients with upper limb fractures following brachial plexus block.

**Methods:**

A total of 149 patients with unilateral upper limb fractures who underwent open reduction and internal fixation surgery under brachial plexus block anesthesia from November 2021 to August 2022 were selected as the subjects for the study and randomly divided into the esketamine group (RNK group) and the ropivacaine group (R group). The incidence of rebound pain at 48 h postoperatively, intraoperative mean arterial pressure (MAP) and heart rate (HR), the onset time and duration of blockade, the Numeric Rating Scale (NRS) scores for pain at rest and with activity during the perioperative period, the dosage, numbers of compressions, and effective compressions of postoperative patient-controlled analgesia with sufentanil, and occurrence of adverse reactions were assessed and compared between the two groups.

**Results:**

The incidence of rebound pain in the RNK group was lower than that in the R group (*P* < 0.05). The RNK group exhibited higher MAP and HR at 5 min and 10 min after anesthesia compared to the R group (*P* < 0.05). The RNK group had faster onset time and longer duration of sensory and motor blockade compared to the R group (*P* < 0.05). The NRS scores at rest and with activity at 12 h and 24 h postoperatively in the RNK group were lower than those in the R group (*P* < 0.05). The total numbers of compressions, effective numbers of compressions, and dosage of sufentanil postoperatively were lower in the RNK group compared to the R group (*P* < 0.05). The incidence of adverse reactions in the RNK group did not differ significantly from that in the R group (*P* > 0.05).

**Conclusions:**

The combination of esketamine and ropivacaine demonstrates a favorable preventive effect on rebound pain in patients with upper limb fractures following brachial plexus block, which is conducive to reducing the incidence of rebound pain, shortening the onset time of blockade, and prolonging the duration of blockade.

**Clinical Trial Registration:**

ClinicalTrials.gov, identifier (ChiCTR2100053035).

## Introduction

Nerve block refers to the injection of local anesthetics around nerve trunks, nerve plexuses, or nerve ganglia, to block their conduction of nerve impulses, thereby achieving an anesthetic effect on their respective innervation areas. Compared to general anesthesia, nerve block only requires injection at a specific site to achieve satisfactory anesthesia, which can reduce the dosage of anesthetic drugs needed and lower postoperative adverse reactions, making it widely used in clinical practice (1, 2). Peripheral nerve block (PNB) is one of the nerve block procedures. Existing research (3) has indicated that the implementation of brachial plexus block anesthesia in patients undergoing surgical treatment for upper arm and forearm fractures can significantly inhibit the neural conduction mediated by the trunks of the brachial plexus, thereby achieving a favorable anesthetic block effect, which not only meets the surgical requirements but also aids in alleviating postoperative pain for patients. Especially in recent years, with the continuous advancement of ultrasound-guided techniques, PNB under ultrasound guidance has demonstrated promising application prospects (4).

However, recent research (5) has also indicated that some patients undergoing PNB may experience a sharp increase in rebound pain following the subsidence of pharmacological effects. Previous research (6) shows that the incidence of postoperative rebound pain in patients undergoing surgical intervention with PNB for wrist and ankle fractures is approximately 35%–41%. A study (7) on patients undergoing day-case surgery with PNB found that the likelihood of experiencing rebound pain within 24 h postoperatively was as high as 49.6% among respondents. Differing from the immediate pain post-general anesthesia, rebound pain occurs at a later time, manifesting as a delayed and sudden increase of rebound pain, with the NRS scores even higher than 7 in some patients. The specific mechanism of rebound pain is currently not thoroughly understood, but undoubtedly, the presence of this phenomenon is detrimental to the patient's perioperative rehabilitation process, and may even potentially lead to doctor-patient conflicts. Therefore, proactive intervention is of great significance (8).

The purpose of this study was to investigate the application of esketamine combined with ropivacaine in upper limb fracture patients and analyze the occurrence of rebound pain after brachial plexus block, thereby providing clinical guidance to improve the perioperative experience of fracture patients.

## Materials and methods

### Study subjects

This is a prospective, double-blind, and randomized controlled study. The study was approved by the Ethics Committee of Xuzhou Cancer Hospital (Approval No. 2021-02-024-K01) and registered with the Chinese Clinical Trial Registry (Registration No.: ChiCTR2100053035). All participants enrolled in this study signed informed consent. Patients with upper limb fractures undergoing open reduction and internal fixation under brachial plexus block anesthesia from November 2021 to August 2022 at Xuzhou Cancer Hospital were selected as the subjects of the study.

Inclusion criteria: (1) patients aged 18−60 years; (2) patients classified as ASA grade I-II (9); (3) patients scheduled for elective open reduction and internal fixation surgery for upper limb fractures in the hospital.

Exclusion criteria: (1) patients with coagulation disorders, systemic or local infections; (2) patients with a history of neurological, psychological, or neuromuscular diseases; (3) those requiring long-term analgesic medication due to chronic pain; (4) bilateral upper limb surgery patients; (5) BMI <18.5 or ≥25.0 kg/m^2^; (6) individuals with multiple injuries requiring other surgeries or the use of alternative analgesics; (7) preoperative nerve injury patients; (8) allergic to the study drugs; (9) patients with poorly controlled hypertension; (10) uncontrollable hyperthyroidism; (11) diabetics; (12) patients with poor treatment compliance; (13) refusal of treatment or nerve block; (14) patients with poor language proficiency, unable to use the NRS scale for pain assessment; (15) patients or relatives refusing to sign the informed consent form; (16) postoperative refusal to use PCIA.

Elimination criteria: (1) patients with incomplete intraoperative nerve block who received adjunctive pharmacotherapy; (2) patients with inadequate intraoperative block effect, necessitating a change in the anesthesia modality; (3) participants requesting withdrawal midway.

### Grouping and randomization

The method of random number table was used to categorize the enrolled patients into the esketamine group (RNK group, receiving ropivacaine combined with esketamine for anesthesia, *n* = 75) and the ropivacaine group (R group, receiving ropivacaine alone for anesthesia, *n* = 74). Patients in the RNK group received 30 ml of 0.375% ropivacaine + 0.5 mg/kg esketamine, while those in the R group received 30 ml of 0.375% ropivacaine. Upon enrollment of patients, the allocation results were disclosed. Patients and anesthesiologists were uninformed about the allocation results. Allocation and medication disposition were performed only by a designated assistant who was subsequently involved in data collection, perioperative follow-up, and outcome assessment.

### Anesthesia methods

Patients enrolled in this study routinely fasted from both food and drink 8 h prior to surgery and did not receive sedation or analgesia before the procedure. During anesthesia preparation, vital signs were routinely monitored. Following the establishment of venous access, ultrasound-guided supraclavicular brachial plexus block was performed. The specific measures were as follows: The patient was placed in a supine position, and after routine disinfection of the puncture site, the ultrasound probe was used to locate the hypoechoic subclavian artery above the first rib. The in plane technique was used to enable that the nerve block needle was inserted parallel to the long axis of the probe, with the needle tip directed towards the position of the nerve bundle. Following successful puncture, 3–5 ml of medication was injected, and then local anesthetic was injected gradually from deep to superficial until the nerve bundle was completely infiltrated. Thirty minutes after successful blockade, the efficacy of the block was assessed by pinprick. During the procedure, continuous oxygen supplementation was provided to the patient, and hemodynamic parameters were monitored. The postoperative analgesic regimen was PCIA (1.0 μg/ml sufentanil + 0.9% NaCl diluted to 200 ml), without a background infusion, with patient-controlled dosage of 5.0 ml per administration, and a lockout interval of 20 min.

## Observation indicators

### Primary observation indicators

At 48 h postoperatively, the incidence of rebound pain was assessed using the Numeric Rating Scale (NRS) to evaluate patients’ pain status. A linear scale of 0 − 10 cm was employed in the NRS (10) to represent the intensity of pain, with 0 signifying absence of pain and 10 denoting severe pain. An NRS score of 7 or higher indicates the presence of rebound pain.

### Secondary observation indicators

(1) Upon entering the operating room, at 5 min, 10 min, 20 min, and 30 min of anesthesia, at skin incision, and at the end of the surgery, mean arterial pressure (MAP) and heart rate (HR) were recorded. (2) The onset and duration of blockade, distinguishing between sensory and motor blockade, were assessed in two patient groups. The onset time of blockade refers to the interval between the completion of anesthetic injection and the moment when the patient experiences no pain (though touch sensation may remain) upon needle stimulation in the blocked area. The duration of sensory blockade is the time from the absence of pain sensation to the presence of pain sensation in the blockade area (11). The onset of motor blockade is the time from the completion of anesthesia injection to the time when the patient is capable of horizontal movement in the blockade area but unable to complete flexion and extension movements. The duration of motor blockade is defined as the time from the onset of blockade to the time when patients are able to move freely in the blockade area (12). (2) The NRS scores at rest and with activity were assessed at the time points of patient entering the operating room, 8 h postoperatively, 12 h postoperatively, 24 h postoperatively, and 48 h postoperatively, respectively. (3) The dosages of sufentanil and the numbers of compressions at 24 and 48 h postoperatively were recorded and compared between the two groups. (4) The occurrence of perioperative adverse reactions such as dizziness, drowsiness, and PONV was recorded in both groups.

### Sample size calculation

According to the previous literature (13), the likelihood of rebound pain following a single PNB was 60.0%, and the expected incidence of rebound pain was approximately 35.0% after receiving proactive intervention. Setting α at 0.05 and power at 80%, calculations using Power Analysis and Sample Size (PASS) 15.0 software revealed approximately 59 cases per group. Accounting for the loss to follow-up rate of 20%, the estimated number of patients per group was approximately 74.

Patients admitted from November 2021 to August 2022 (total of 174 cases) were screened according to inclusion and exclusion criteria, with 149 patients ultimately enrolled, comprising 75 cases in the RNK group and 74 cases in the R group.

### Statistical methods

In this study, Statistical Package for the Social Sciences (SPSS) 26.0 was utilized for data collation and statistical analysis, and Graph Prism 9.5.1 was employed for plotting the graphs. Measurement data in the study conformed to normal analysis and were expressed in the form of mean ± standard deviation, with independent samples *t*-tests for comparisons between groups and repeated measures Analysis of Variance (ANOVA) for comparisons of different time points within groups. Categorical data in the study were expressed as rates and tested using the chi-square test for differences between groups. *P* < 0.05 was taken as statistically significant difference.

## Results

### Comparison of baseline clinical data between the two groups of patients

**Figure 1:**
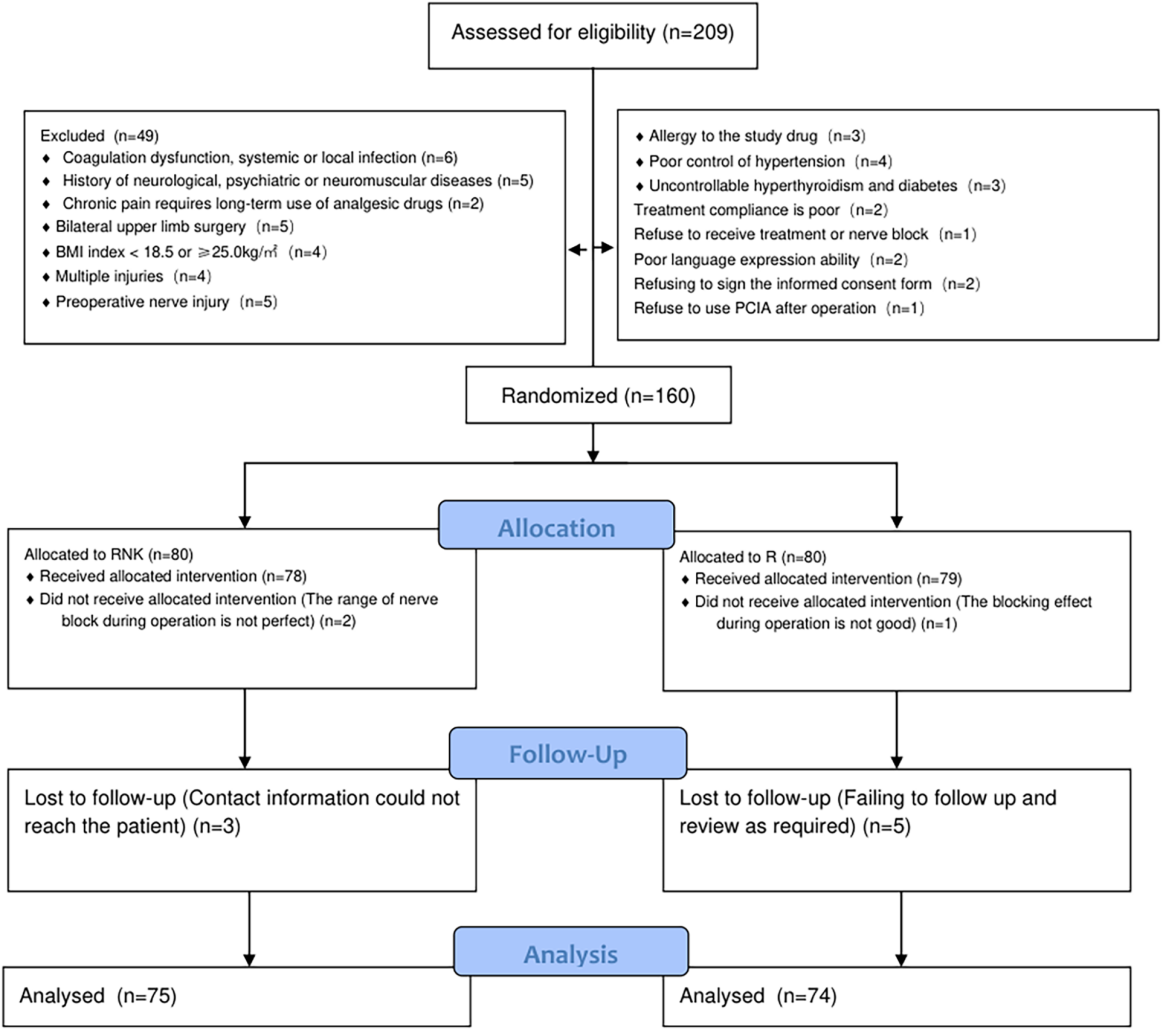
Flow diagram of the specific design.

The baseline clinical data of patients in both groups, including gender, age, body mass index (BMI), operative time, ASA classification, and surgical type, were included and compared, and the results indicated no statistically significant difference between the two groups in the aforementioned data (*P* > 0.05), suggesting good comparability (Table 1). The flow diagram of the specific design is shown in Figure 1.

**Table 1:** Comparison of baseline clinical data between the two groups of patients ($\bar{x}\pm s$)/[*n* (%)].

General clinical data		RNK group (*n* = 75)	R group (*n* = 74)	*t/χ^2^*	*P*
Gender	Male	54 (72.00)	49 (66.22)	0.584	0.445
	Female	21 (28.00)	25 (33.78)		
Average age (years)		43.33 ± 11.32	46.73 ± 10.37	1.818	0.069
Average BMI (kg/m^2^)		22.67 ± 1.71	23.02 ± 1.57	1.279	0.201
Operative time (min)		89.53 ± 28.59	85.81 ± 21.21	0.267	0.790
ASA classification	ASA I	18 (24.00)	17 (22.97)	0.022	0.882
	ASA II	57 (76.00)	57 (77.03)		
Surgical type	Clavicle fracture	39 (52.00)	39 (52.70)	0.862	0.973
	Radial fracture	15 (20.00)	18 (24.32)		
	Ulnar fracture	2 (2.67)	2 (2.70)		
	Metacarpal fracture	8 (10.67)	7 (9.46)		
	Humeral fracture	10 (13.33)	7 (9.46)		
	Phalangeal fractures	1 (1.33)	1 (1.35)		

### Comparison of perioperative MAP between the two groups of patients

**Figure 2:**
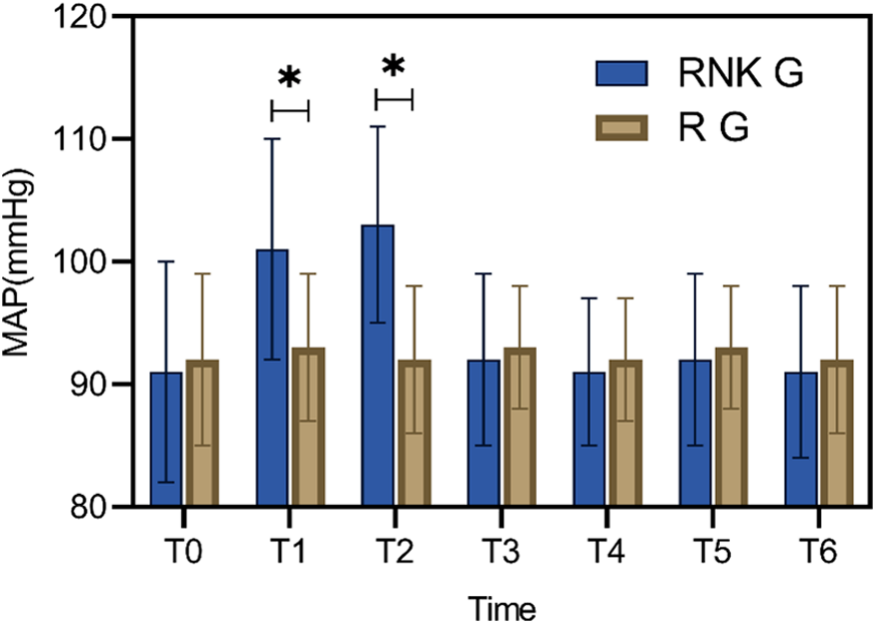
Difference in perioperative MAP between the two groups of patients. At 5 min and 10 min of anesthesia, MAP in the RNK group was higher than that in the R group (*P* < 0.05). * denotes a statistically significant difference between groups.

Patients in the RNK group exhibited higher MAP at 5 min and 10 min after anesthesia compared to upon entering the operating room, 20 min of anesthesia, 30 min of anesthesia, skin incision, and the end of surgery (*P* < 0.05). Furthermore, at 5 min and 10 min after anesthesia, MAP in the RNK group was higher than that in the R group (*P* < 0.05). However, there were no statistically significant differences in MAP between the two groups at other time points (*P* > 0.05) (Figure 2).

### Difference in perioperative HR between the two groups of patients

**Figure 3:**
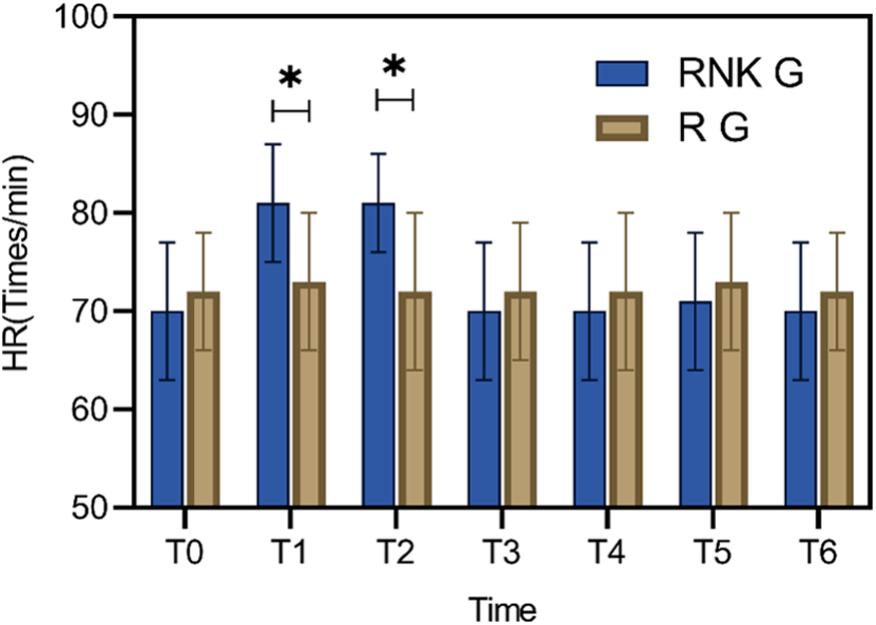
Difference in perioperative HR between the two groups of patients. At 5 min and 10 min after anesthesia, HR in the RNK group was higher than that in the R group (*P* < 0.05). * denotes a statistically significant difference between groups.

Patients in the RNK group exhibited higher HR at 5 min and 10 min after anesthesia compared to upon entering the operating room (*P* < 0.05). Furthermore, at 5 min and 10 min after anesthesia, HR in the RNK group was higher than that in the R group (*P* < 0.05). However, there were no statistically significant differences in HR between the two groups at other time points (*P* > 0.05) (Figure 3).

### Difference in onset and duration of blockade between the two groups of patients

**Figure 4:**
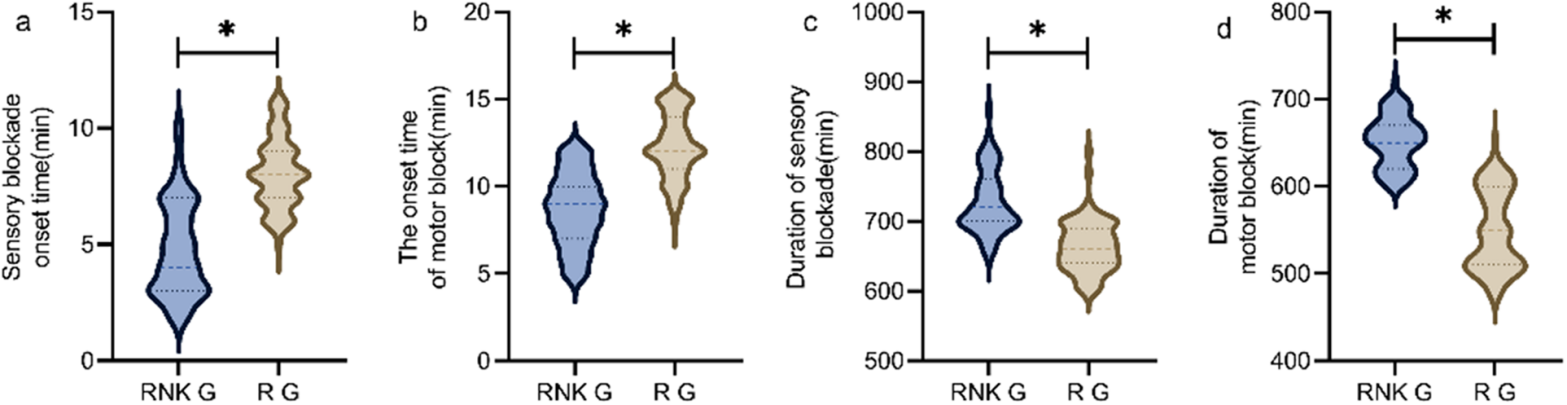
Difference in onset and duration of blockade between the two groups of patients. The RNK group had faster onset time of sensory **(a)** and motor **(b)** blockade compared to the R group, and had longer duration of sensory **(c)** and motor **(d)** blockade compared to the R group (*P* < 0.05). * denotes a statistically significant difference between groups.

The RNK group had faster onset time and longer duration of sensory and motor blockade compared to the R group, showing statistically significant differences between the two groups (*P* < 0.05) (Figure 4).

### Difference in NRS scores at different time points between the two groups of patients

**Figure 5:**
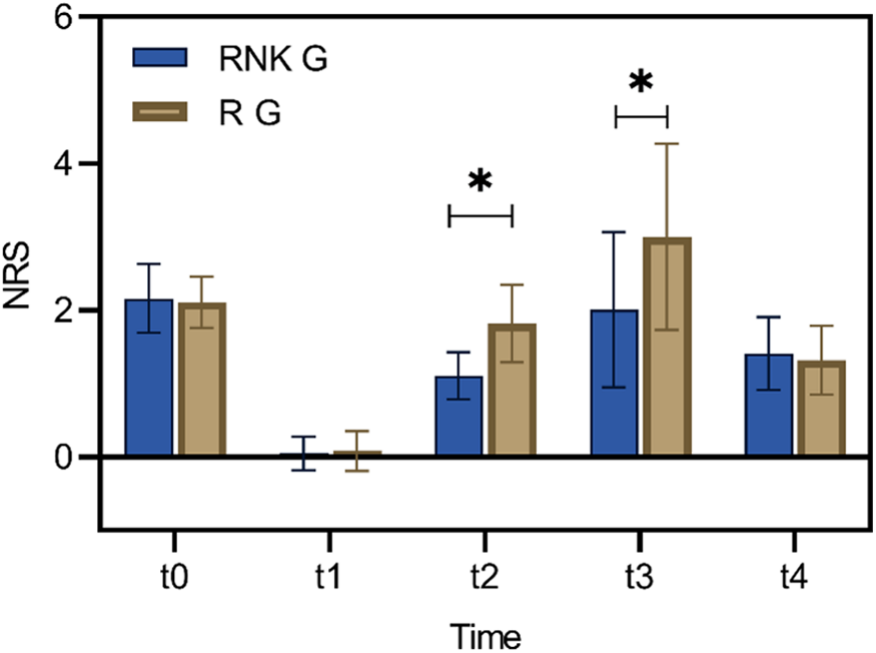
Difference in NRS scores at rest at different time points between the two groups of patients. The NRS scores at rest at t2 and t3 in the RNK group were lower than those in the R group, exhibiting statistically significant difference between the two groups (*P* < 0.05). * denotes a statistically significant difference between groups.

**Figure 6:**
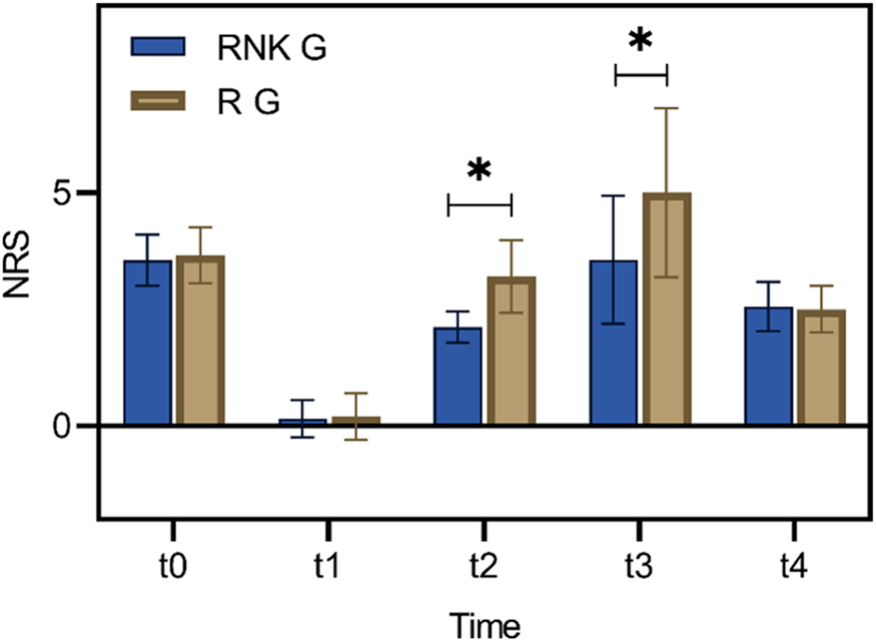
Difference in NRS scores with activity at different time points between the two groups of patients. The NRS scores with activity at t2 and t3 in the RNK group were lower than those in the R group, exhibiting statistically significant difference between the two groups (*P* < 0.05). * denotes a statistically significant difference between groups.

There were no statistically significant difference in NRS scores at rest and with activity upon entering the operating room, 8 h postoperatively, and 48 h postoperatively between the two groups (*P* > 0.05). The NRS scores at rest at 12 h and 24 h postoperatively in the RNK group were lower than those in the R group, exhibiting statistically significant difference between the two groups (*P* < 0.05) (Figure 5). The NRS scores with activity at 12 h and 24 h postoperatively in the RNK group were lower than those in the R group, exhibiting statistically significant difference between the two groups (*P* < 0.05) (Figure 6).

### Comparison of postoperative occurrence of rebound pain between the two groups of patients

**Figure 7:**
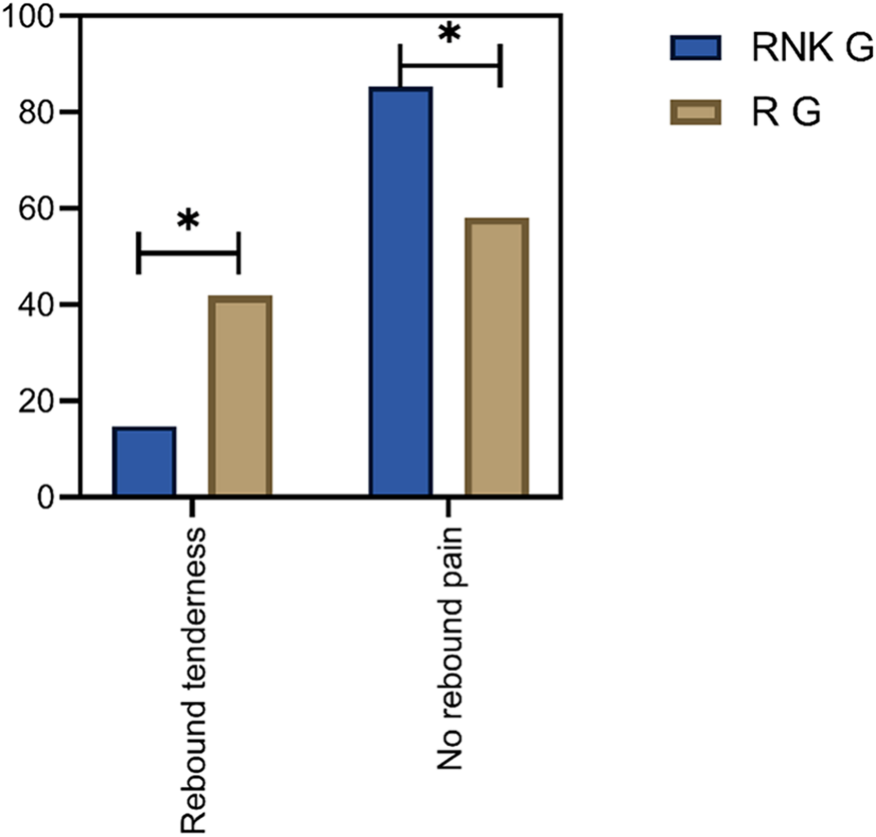
Comparison of postoperative occurrence of rebound pain between the two groups of patients. The incidence of rebound pain in the RNK group was lower than that in the R group (*P* < 0.05).

In the RNK group, 11 patients experienced postoperative rebound pain, with an incidence rate of 14.67%, and the first case of rebound pain occurred 13 h postoperatively. In the R group, 31 patients experienced postoperative rebound pain, with an incidence rate of 41.89%, and the first case appeared 14 h postoperatively. The difference in the incidence of rebound pain was statistically significant between the two groups (*P* < 0.05) (Figure 7).

### Comparison of dosages of sufentanil and numbers of compressions at 24 and 48 h postoperatively

**Figure 8:**
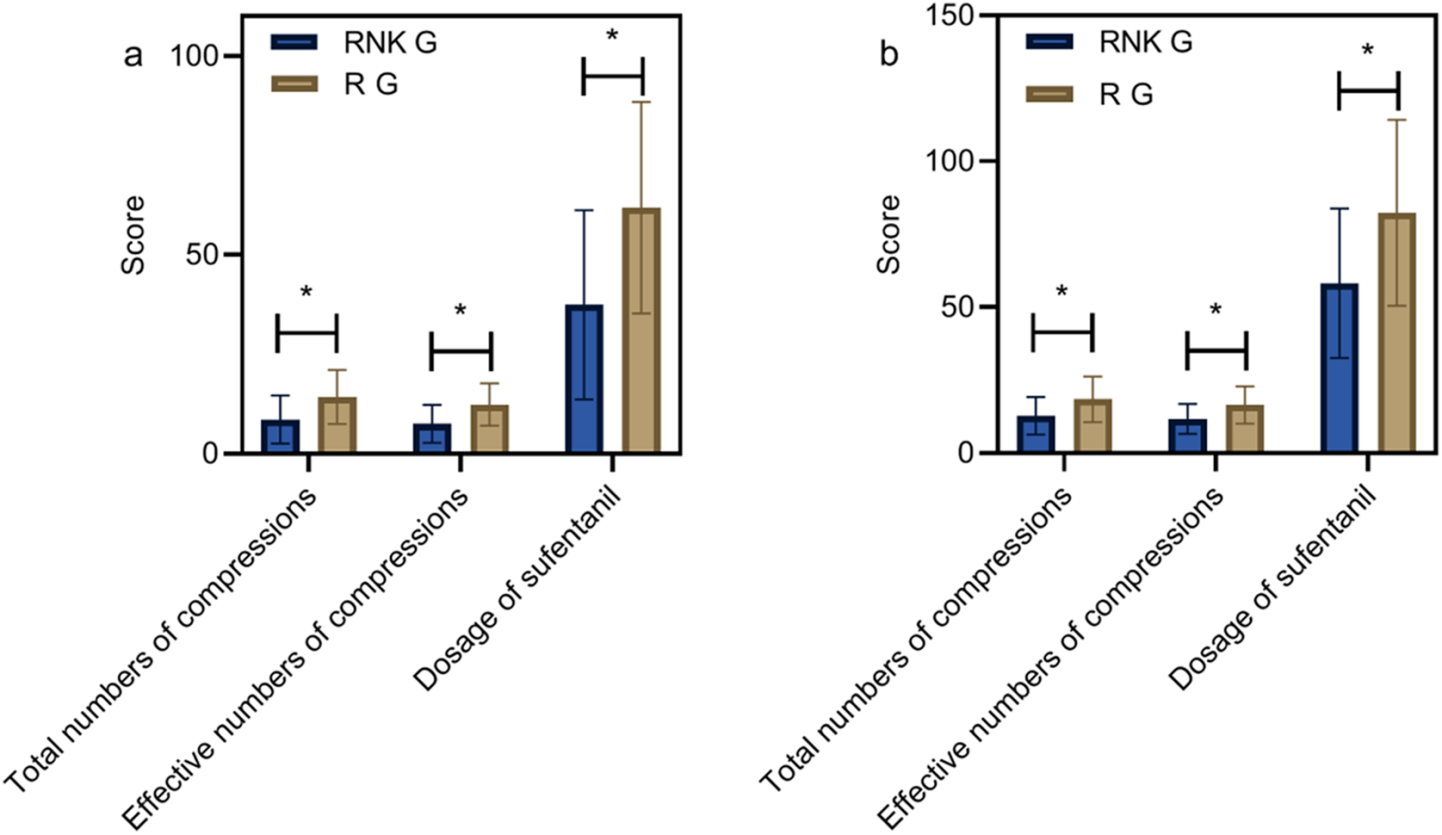
Comparison of dosages of sufentanil and numbers of compressions at 24 and 48 h postoperatively. At 24 h **(a)** and 48 h **(b)** postoperatively, the total numbers of compressions, effective numbers of compressions, and dosage of sufentanil were lower in the RNK group compared to the R group, with statistical significance (*P* < 0.05). * denotes a statistically significant difference between groups.

At 24 h postoperatively, the total numbers of compressions, effective numbers of compressions, and dosage of sufentanil were lower in the RNK group compared to the R group, with statistical significance (*P* < 0.05) (Figure 8a). At 48 h postoperatively, the total numbers of compressions, effective numbers of compressions, and dosage of sufentanil were lower in the RNK group compared to the R group, with statistical significance (*P* < 0.05) (Figure 8b), as shown in Figure 8.

### Comparison of incidence of adverse reactions between the two groups of patients

**Figure 9:**
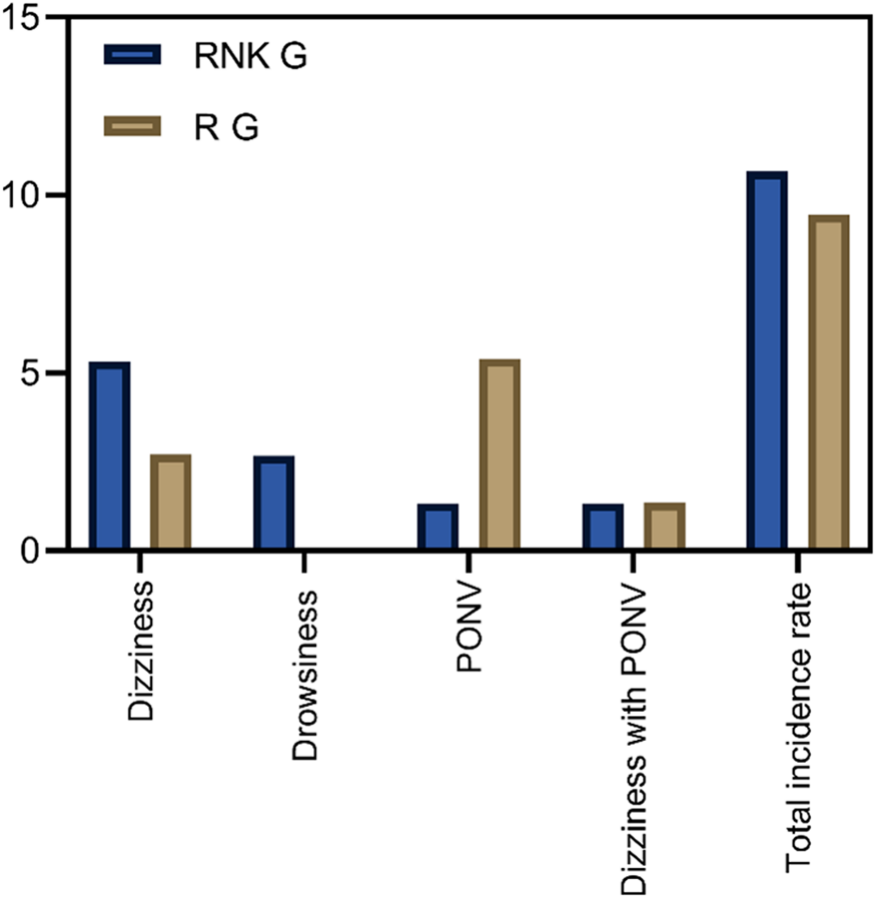
Comparison of incidence of adverse reactions between the two groups of patients. The incidence of adverse reactions in the RNK group (10.67%, 8/75) did not differ significantly from that in the R group (9.46%, 7/74) (*P* > 0.05).

Patients in the RNK group exhibited dizziness in 1 case, drowsiness in 2 cases, PONV in 1 case, and dizziness accompanied by PONV in 1 case. The overall incidence of adverse reactions was 10.67% (8/75) in the RNK group, which did not significantly differ from 9.46% (7/74) in the R group (*P* > 0.05) (Figure 9).

## Discussion

The findings of this study suggest that the use of esketamine in combination with ropivacaine for brachial plexus block in patients with upper limb fractures is effective in preventing postoperative rebound pain, while also shortening the onset time of the blockade and prolonging its duration.

Postoperative pain is one of the common clinical issues during the perioperative period of surgical procedures, and its occurrence is closely associated with the patient's psychological factors, cardiopulmonary complications, postoperative rehabilitation exercises, preoperative chronic pain, etc. The emergence of pain significantly impacts the patient's postoperative rehabilitation process and even has a certain correlation with postoperative mortality rates (14). In clinical practice, opioid analgesics have commonly been employed for pain management in patients. In recent years, multiple studies (15, 16) have confirmed the superior efficacy of combined PNB in alleviating perioperative pain associated with surgical procedures. However, clinical practice has also pointed out that PNB has several drawbacks, such as its limited duration of action; following the diminishing effects of nerve block medication, patients often experience pain rebound phenomenon, which seriously affects the postoperative sleep quality and necessitates increased dosage of opioid analgesics (17). The mechanism underlying rebound pain after nerve block is currently unclear, but existing research (18) suggests that factors such as age, surgical site, type of surgery, protocol of surgery, nerve injury, and local anesthetic drug concentration may all potentially influence the occurrence of this phenomenon. For instance, the phenomenon exhibits a lower incidence in individuals >60 years of age, a relatively higher incidence in younger patients, a lower incidence in knee surgeries relative to shoulder surgeries (The cause may be related to the neural innervation of the knee and shoulder joints. The shoulder joint is innervated by the brachial plexus, with numerous nerve branches and a wide sensory range, making it prone to rebound pain after the anesthetic wears off. In contrast, the knee joint is primarily innervated by the femoral and sciatic nerves, with a relatively smaller and less complex nerve distribution), and a higher incidence in younger female patients than in other individuals (10).

This study analyzed the clinical value of esketamine combined with ropivacaine for brachial plexus block anesthesia in patients with upper limb fractures through a randomized controlled trial, and the results showed that compared to patients in the R group who underwent block anesthesia with ropivacaine alone, patients in the RNK group receiving esketamine combined with ropivacaine exhibited significantly shorter onset of sensory and motor blockade and longer duration of sensory and motor blockade, similar to findings in other literature. These results suggested that ropivacaine combined with esketamine could shorten the onset time of block and prolong the duration of blockade. The reasons may be as follows: Esketamine belongs to the class of NMDA receptor antagonists, and its mechanism of action primarily involves reducing the release of excitatory amino acids and exerting analgesic effects on various opioid receptors. The combination of esketamine and ropivacaine exhibits a synergistic effect, hastening the onset of anesthesia, and the combined use of these two drugs also prolongs the duration of blockade (19). However, the findings of some scholars contradict this study, as demonstrated by Touil et al. (20), which has indicated that adding 0.3 mg/kg of ketamine on the basis of conventional nerve block does not decrease the occurrence rate of postoperative rebound pain in patients. The reasons for the aforementioned differences may be as follows: (1) variance in medication, the study of above-mentioned scholars used ketamine for control experiments, whereas our study opted for esketamine, the dextrorotatory structure of ketamine with stronger potency; (2) difference in administration method, the above-mentioned scholars chose intravenous administration, whereas our study opted for perineural injection; esketamine perineural injection can bind to opioid receptors, exerting inhibitory neural conduction effects, while ketamine intravenous injection merely exhibits local anesthetic effects, and comparatively, esketamine demonstrates superior nerve block efficacy (21).

The study also conducted follow-up on the perioperative pain intensity of enrolled patients, indicating that both NRS scores at rest and with activity of patients in the RNK group at 12 h and 24 h postoperatively were lower than those in the R group, and the postoperative incidence of rebound pain was significantly lower in the RNK group (14.67% vs. 41.89%). The results suggested that the addition of esketamine prolonged the duration of nerve block by approximately 12 h compared to the administration of ropivacaine alone, maintaining it for a minimum of 24 h, which is in line with the research findings of other scholars (22). From the horizontal analysis of NRS scores in this study, it could be observed that the time period from 12 h–24 h postoperatively was when the intensity of patients’ pain increased, and even with the addition of esketamine, the trend in NRS score changes for patients in the RNK group was similar to that of the R group. This phenomenon may serve as a reference for subsequent research. Whether this trend can be changed or even reversed by increasing the dosage of esketamine to further reduce patients’ postoperative pain intensity of needs to be demonstrated in further research.

The perioperative MAP and HR of the two groups of patients were also compared, and the results suggested that at 5 min and 10 min of anesthesia, patients in the RNK group had higher HR and MAP values than those in the R group. This phenomenon may be attributed to the influence of esketamine on the reuptake of catecholamines (23). In spite of this phenomenon, subsequent comparisons at 20 min and 30 min of anesthesia revealed no statistical difference in MAP and HR values between the two groups, suggesting that the administration of esketamine does not significantly impact patients’ hemodynamic parameters (24), and its safety is worthy of affirmation, which is also confirmed by the comparison of adverse reactions between the two patient groups in this study.

## Conclusion

The combination of esketamine and ropivacaine demonstrates a favorable preventive effect on rebound pain in patients with upper limb fractures following brachial plexus block, which is conducive to reducing the incidence of rebound pain, shortening the onset time of blockade, prolonging the duration of blockade, and reducing the postoperative dosage of sufentanil. The limitations of this study are as follows: (1) The single dosage of esketamine was used, and whether different dosages of esketamine affect the severity of perioperative pain in patients were not investigated. (2) Patient follow-up lasted only 48 h, without tracking whether the addition of esketamine would impact long-term effects on patient experience. These aforementioned issues are intended to be further elucidated in subsequent research endeavors.

## Data Availability

The original contributions presented in the study are included in the article/Supplementary Material, further inquiries can be directed to the corresponding author.
